# Case Report: *Mycobacterium senegalense* Infection After Cholecystectomy

**DOI:** 10.3389/fpubh.2022.899846

**Published:** 2022-07-11

**Authors:** Huiling Zhou, Hong Yang, Fengling Gong, Shaolong Zhou, Yifeng Yang, Haidan Liu, Jijia Liu

**Affiliations:** ^1^Department of Cardiovascular Surgery, The Second Xiangya Hospital, Central South University, Changsha, China; ^2^Clinical Center for Gene Diagnosis and Therapy, The Second Xiangya Hospital, Central South University, Changsha, China; ^3^Department of General Surgery, Xiangyin People's Hospital, Yueyang, China; ^4^Department of Anesthesiology, Xiangdong Hospital Hunan Normal University, Zhuzhou, China; ^5^Hengyang Medical School, University of South China, Hengyang, China

**Keywords:** *Mycobacterium senegalense*, infection, non-tuberculous mycobacterium, metagenomic next-generation sequencing, case report

## Abstract

**Background:**

*Mycobacterium senegalense* is a non-tuberculous mycobacterium and is found everywhere in the environment. However, *M. senegalense* infection in human is extremely rare, especially in immunocompetent individuals. It is difficult to detect *M. senegalense* infection because its symptoms are non-specific, and routine diagnostic tests are less sensitive. It is also resistant to commonly used antibiotics. Here, we report the first case of *M. senegalense* infection after laparoscopic cholecystectomy in China.

**Case Presentation:**

A 55-year-old man was admitted because of repeated infections at multiple incision sites for more than 1 year. Although routine diagnostic test results were negative, metagenomic next-generation sequencing (mNGS) identified DNA sequences of *M. senegalense* in tissue samples from incision sites. The presence of *M. senegalense* was further confirmed by polymerase chain reaction and capillary electrophoresis. After 60 days of quadruple therapy with clarithromycin, moxifloxacin, rifampicin, and oxycycline, the patient's wound healed.

**Conclusion:**

We believe the case findings contribute to the limited amount of knowledge about *M. senegalense* infection and raises awareness that this infection can result in poor wound healing, even in an immunocompetent host. Owing to a lack of early, precise diagnosis, it is difficult to treat *M. senegalense* infections. Based on our findings, mNGS is a sensitive diagnostic test for *M. senegalense* infections.

## Introduction

*Mycobacterium senegalense* is a non-tuberculous mycobacterium (NTM) that was first reported to cause bovine farcy in sub-Saharan Africa, exhibiting chronic granulomatous inflammation of skin lymphatics and draining lymph nodes of zebu cattle (1). However, human infections with *M. senegalense* are rare, and its zoonotic potential is unknown.

NTM is a complex pathogen that is usually a commensal or saprophytic organism. It is extensively found in the environment such as in soil and water sources, leading to high human–pathogen exposure (2), although only a few species infect humans. NTM is an opportunistic pathogen that usually infects immunocompromised patients with intractable conditions such as those with underlying lung diseases, human immunodeficiency virus infections, and cancers, as well as those receiving chemotherapy (3). NTM can be divided into two groups based on how long they take to grow in a culture: rapid-growing and slow-growing species. The most common pathogen among the slow-growing NTM species is *M. avium-intracellulare*, which primarily causes pulmonary infection (4). *M. fortuitum* is the most common pathogen among the rapid-growing NTM species, which usually causes colonized or transient infections in the respiratory tract, in addition to disseminated disease, lymphadenitis, and skin and soft tissue infections (5, 6). Although *M. senegalense* belongs to the same group as *M. fortuitum*, it rarely infects humans.

So far, only seven cases of humans being infected by *M. senegalense* have been described; however, none of them appear to have occurred in China. In this study, we report the first case of *M. senegalense* infection after laparoscopic cholecystectomy in China. mNGS test identified that the patient was infected with *M. senegalense*, even though routine diagnostic tests were negative. The patient's condition improved, and the wound healed after quadruple therapy with clarithromycin, moxifloxacin, rifampicin, and doxycycline.

## Manuscript Formatting

### Case Presentation

**Figure 1:**
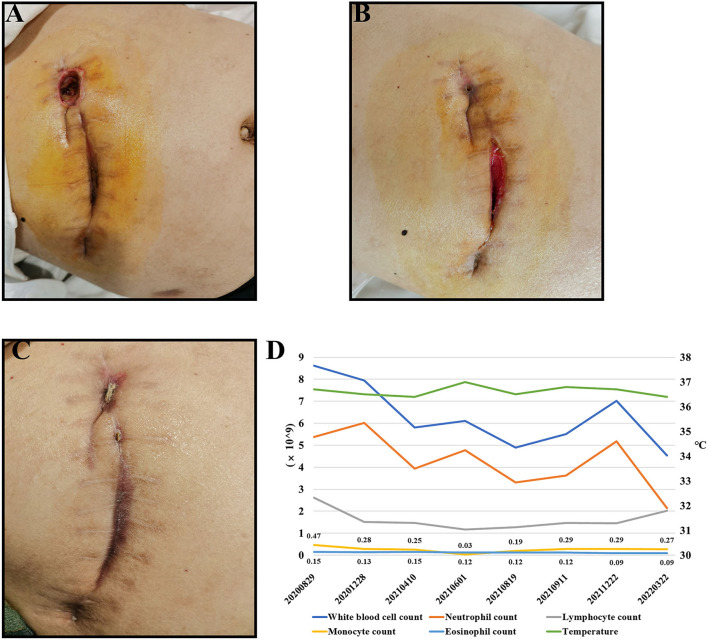
Condition of the patient's incisions. **(A)** It shows the incisions of the patient on admission. **(B)** It shows the incisions of the patient after second surgery. **(C)** It shows the patient's incisions after 60 days of quadruple therapy. **(D)** The patient's temperature and routine blood test results.

A 55-year-old male patient, who present to our hospital in August 2021, because of repeated infection at multiple incisions for more than 1 year after laparoscopic cholecystectomy surgery done for gallstone complicated with cholangitis. On examination, the incisions under the xiphoid process were red and swollen, the incisions were dehiscent, local tenderness, high skin temperature and a little suppuration (Figure 1A).

**Figure 2:**
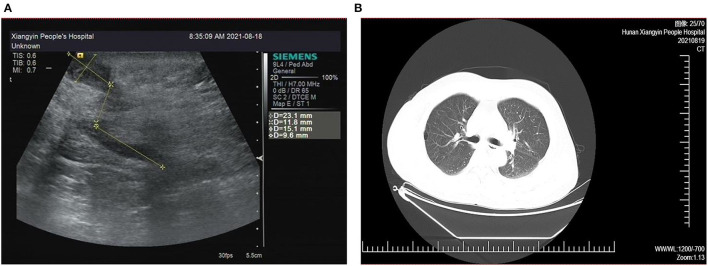
**(A)** Abdominal B-ultrasound results show the formation of sinus tract and empyema. **(B)** Chest computed tomography images of the lung window show no sign of tuberculosis-related diseases.

The patient had multiple incisions infections and repeated purulent exudates after surgery, with an average recurrence about 25 days, but the blood routine results shown no abnormality. According to the B-ultrasound results (Figure 2A), considering the formation of sinus tract under xiphoid process. In September 2021, laparoscopic re-surgery and abdominal exploration were performed, the sinus trace was cut and drainage of abscess. Then given piperacillin sodium and tazobactam sodium anti-infection treatment, but the effect was still poor after a period of time. We discovered the surgical incisions still were not healing, the skin surrounding the incisions was red, swollen and painful, and suppuration was present (Figure 1B).

Since admission, the patient's body temperature and blood routine results were normal (Figure 1D). Additionally, regular bacterial culture and acid-fast bacillus (AFB) staining were negative, but blood samples for the tubercle bacillus antibody (TB-Ab) test were positive. Combined with the patient's chest CT (Figure 2B), tuberculosis related examination and the patient's clinical symptoms, they did not support tuberculosis-related diseases. In order to further resolve the question and determine the pathogens, the mategenomic sequencing technology covering the pathogen is used to accurately identify pathogens.

**Figure 3:**
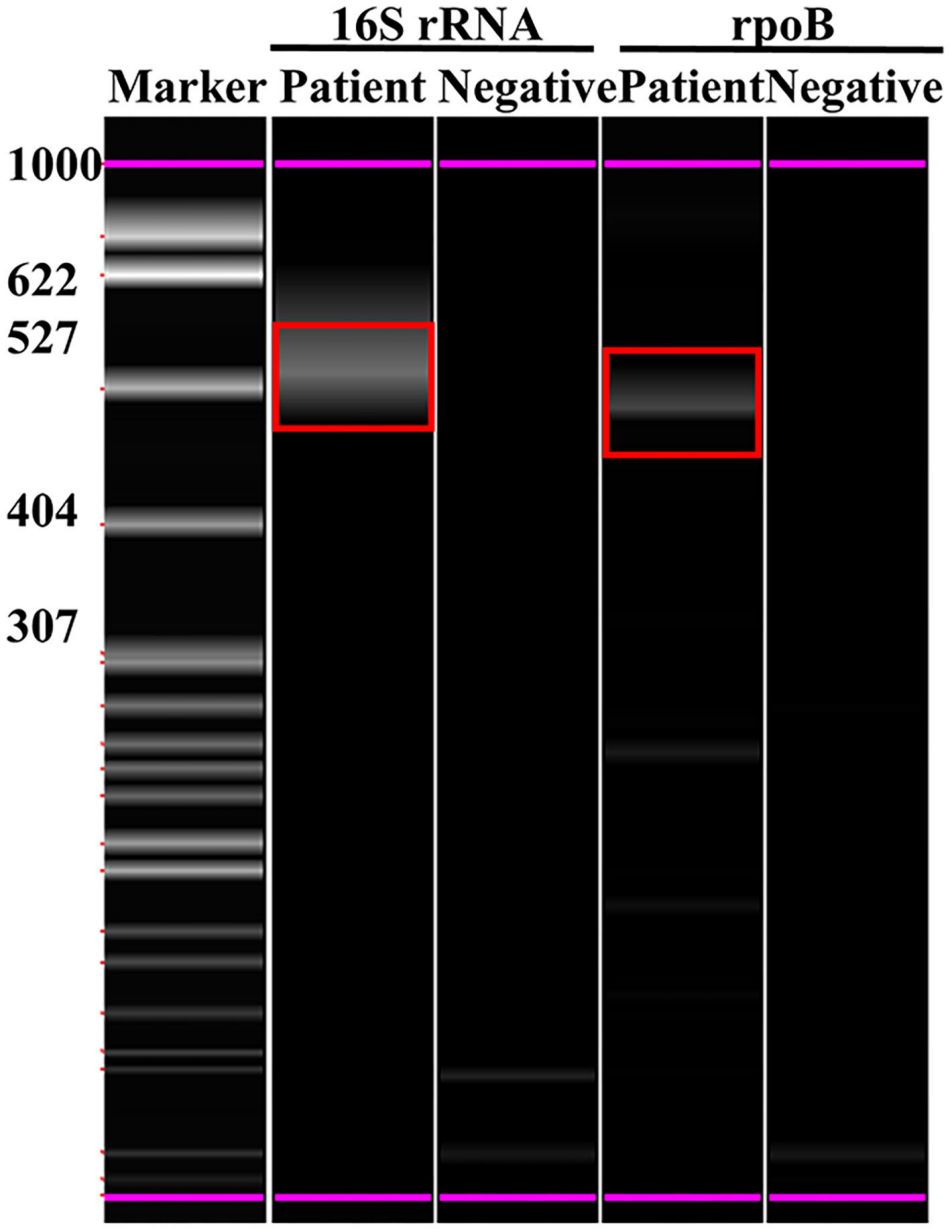
Polymerase chain reaction and capillary electrophoresis technique confirmed *M. senegalense* infection in the patient. 16S rRNA- and *rpoB*-specific amplified fragments are marked with red boxes.

On November 29, 2021, the tissue from the incisions of the patient was sampled for DNA metagenomic next-generation sequencing (mNGS) (KingMed Diagnostics, Changsha, China). It detected 53 sequences that could be mapped to *M. senegalense* in a total of 113 sequences, and the coverage was 0.09%, making up 58.76% of the total microbe sequences (Table 1). Targeted PCR of *M. senegalense* using two pairs of primers was applied: 16S RNA forward 5′-AGCGGCGGAGCATGTGGATTA-3′, reverse 5′-GCTGATCTGCGATTACTAGCGACTC-3′ (GenBank: DQ145802.1); rpoB forward 5′-TGCGTGCCATCTTCGGTGAGA-3′, reverse 5′-GTCGATGTTCCAGCCTGCCTTG-3′ (GenBank: JF706631.1). The primers were designed and verified using Primer-BLAST based on the reference genome sequence of *M. senegalense* in NCBI. Subsequently, the capillary electrophoresis technique (Qsep 100TM; Bioptic) also curtained the *M. senegalense* infection (Figure 3).

**Table 1:** mNGS results of the tissue and exudate from the punctures in this case.

**Genus**			**Species**	
**Generic** **name**	**Relative** **abundance (%)**	**Number of sequences**	**Species** **name**	**Number of sequences**
*Mycobacterium*	58.76%	113	*Mycobacterium senegalense*	53

According to the results, the patient was initiated with oral clarithromycin (500 mg, twice daily), moxifloxacin (400 mg, once daily), rifampicin (450 mg, once daily) and doxycycline (100 mg, once every 12 hours). Then the swelling gradually subsided and there was no obvious purulent exudation. After 20 days, the patient's incisions healed well, and there was no sign of recurrence in the 60th day after quadruple therapy (Figure 1C).

## Discussion

We present a unique case of a patient with chronic incision site infections who underwent laparoscopic cholecystectomy and re-surgery, after which he was diagnosed as having an infection caused by an unusual NTM species.

Rapid-growing NTM species, such as *M. fortuitum*, have increasingly gained recognition during the last two decades because of their ability to thrive even in the harshest environments. In an immunosuppressed patient, these pathogens cause serious infections, such as bacteremia (5). *M. senegalense* is a rapid-growing NTM, belongs to the same group as *M. fortuitum*, and was first isolated from a bovine source (6). Infection with *M. senegalense* affects the sinus tracts and is characterized by multiple abscesses and granuloma formation. Only 7 cases about human infection with *M. senegalense* have been reported, the recent report found it to cause skin infections in immunocompetent patients (7). Most patients had minimal history of contact with animals and travel to areas where *M. senegalense* infection was more commonly endemic (8–10). And most of those cases were identified by various sequencing techniques. The first case of human infection by *M. senegalense* was described in 2005 (11). The patient had non-Hodgkin's lymphoma and was treated with R-CHOP. Her physical examination revealed high fever of 39.8°C, although she had no contact with any animal- or bovine-specific sources. Although it was difficult to diagnose, 16S rRNA gene, *rpoB*, and 16S-23S rRNA gene internal transcribed spacer sequence analyses finally revealed *M. senegalense* infection. Another case reported *M. senegalense* infection in a healthy girl with no prior history of serious illness or hospitalization (12). The patient was scratched by fish tank debris, following which the wound was sterilized and sutured. After 2 weeks, the wound failed to heal and appeared reddened and indurated. 16S gene sequencing and biochemical testing was used to diagnose the *M. senegalense* infection, which was the first infection reported in humans without immunodeficiency. This also indicated that immunocompetent individuals were also at a risk of infection.

It is worth mentioning that the patient had no history of contact with animals, and the patient's body temperature, routine blood test results, and other data indicated that infection was absent. These indicators were not present in previous cases, suggesting that *M. senegalense* may not cause changes in infection indicators. According to current literature, the prevalence and incidence of NTM infections continue to increase (11, 13). Rapid identification and subsequent drug susceptibility testing are essential for sleeting appropriate antibiotics against NTM. In addition, it is necessary to be vigilant about NTM infections, even if individuals have no contact history with animals, no travel history, and no infection-negative routine blood test results with recurrent infections at laparoscopic incision sites.

Because of the scarcity of literature on *M. senegalense*, we draw on treatment guidelines for NTM infections (14, 15). From the literature review, we found that the management of NTM infections is quite difficult. First, NTM infections possess several internal antimicrobial resistance mechanisms such as an impermeable cell wall and formation of granulomas, which reduces antimicrobial influx. Second, the long-term treatment of NTM infections leads to the development of drug resistance (16). According to the American Thoracic Society, Infectious Diseases Society of America, and Clinical and Laboratory Standards Institute, the latest recommended NTM drugs include macrolides, clofazimine, quinolones, sulfonamides, linezolid, aminoglycosides, bedaquiline, and tetracyclines (17). Additionally, the success rate of treatment for NTM infection is species specific. Usually, a combination of three or four drugs are administered to avoid the development of resistance to monotherapy. Based on previous experience combined with this patient's condition, we administered clarithromycin (500 mg, twice daily), moxifloxacin (400 mg, once daily), rifampicin (450 mg, once daily), and doxycycline (100 mg, once every 12 h). With this combination of therapy, the patient achieved good clinical efficacy. The case report provides a reference for the treatment of *M. senegalense*.

## Data Availability Statement

The original contributions presented in the study are included in the article/Supplementary Material, further inquiries can be directed to the corresponding authors.

## Ethics Statement

Written informed consent was obtained from the individual(s) for the publication of any potentially identifiable images or data included in this article.

## Author Contributions

HY, FLG, and SLZ collected and analyzed patient data. HLZ, HY, YFY, and HDL wrote the manuscript. HDL and JJL provided supervision. All authors reviewed, edited, and approved the final manuscript.

## Funding

This study was supported by National Natural Science Foundation of China (82073260 to HDL).

## Conflict of Interest

The authors declare that the research was conducted in the absence of any commercial or financial relationships that could be construed as a potential conflict of interest.

## Publisher's Note

All claims expressed in this article are solely those of the authors and do not necessarily represent those of their affiliated organizations, or those of the publisher, the editors and the reviewers. Any product that may be evaluated in this article, or claim that may be made by its manufacturer, is not guaranteed or endorsed by the publisher.
