# Supplementary material for: Case Report: Mycobacterium senegalense Infection After Cholecystectomy
Source: Front Public Health. 2022 Jul 11;10:899846. doi: 10.3389/fpubh.2022.899846 (PMC9309716; doi:10.3389/fpubh.2022.899846)
Supplement: Supplementary file 1 [file Data_Sheet_1.DOCX]

Supplementary Materials and Methods

# mNGS

## Sample

The samples from incisions’ tissue were stored and transported in dry ice, the whole of process of pathogen detection pipeline and capillary electrophoresis were carried out in the laboratory of Changsha Kingmed Medical Test Center Co.Ltd..

## DNA Extraction and Quality Control

The DNA was extracted by QIAsymphony Circulating NA Kit (Cus.48), along with a negative control and a positive control. Then, using TruePrepTM DNA Library Prep Kit V2 for Illumina® to prepare the DNA libraries and using Qubit to measure the libraries concentration. As well as the sequencing was carried out by Illumina nextseq 500 system with 75 cycles Reagent Kit.

Then, filtering out low-quality sequencing data, removing the sequences mapped to human reference genome and aligning the remaining data to the microbial genome database. All the process was similar to that described in a previous published mNGS article which was conducted in Guangzhou Kingmed Medical Test Center Co.Ltd.(1).

## Bioinformatic Analysis

The microbial genome database is MetagenomicX for clinical usage. It contains 36497 microorganisms’ genomes, which covers most of the microbial genomes which have been sequenced. And 8704 of the total microorganisms in the first-grade database have integral sequence of whole genome and detailed clinical analysis, which covers most of the known pathogenic bacteria, viruses, fungi and parasites. This first-grade database with high-quality genomes is used as first choice. Those has been described in another published mNGS article which was conducted in Changsha Kingmed Medical Test Center Co.Ltd. (2).

1. Shi Y, Chen J, Shi X, Hu J, Li H, Li X, et al.(2021). A case of chlamydia psittaci caused severe pneumonia and meningitis diagnosed by metagenome next-generation sequencing and clinical analysis: a case report and literature review. *BMC Infect Dis* 21: 621.doi:10.1186/s12879-021-06205-5.

2. Lei C, Zhou X, Ding S, Xu Y, Yang B, Guo W, et al.(2022). Case Report: Community-Acquired Legionella gormanii Pneumonia in an Immunocompetent Patient Detected by Metagenomic Next-Generation Sequencing. *Front Med (Lausanne)* 9: 819425.doi:10.3389/fmed.2022.819425.
